# Synergistic Regulatory Effects of Water–Nitrogen Coupling on Osmotic Regulation, Yield, and Forage Quality of Alfalfa

**DOI:** 10.3390/plants15020173

**Published:** 2026-01-06

**Authors:** Yi Ling, Minhua Yin, Yanxia Kang, Guangping Qi, Yanlin Ma

**Affiliations:** College of Water Conservancy and Hydropower Engineering, Gansu Agricultural University, Lanzhou 730070, China; 1073324120786@st.gsau.edu.cn (Y.L.); yanxiakang@126.com (Y.K.); qigp@gsau.edu.cn (G.Q.); mayl@gsau.edu.cn (Y.M.)

**Keywords:** water–nitrogen coupling, osmotic adjustment, yield, quality, alfalfa

## Abstract

Water scarcity and poor soil fertility are major limiting factors constraining agricultural production in the arid and semi-arid regions of Northwest China. Water–nitrogen synergistic regulation is an important approach to improving crop growth and enhancing agricultural productivity. In this study, four irrigation levels—severe water deficit (W1: 45–65% θ_f_), moderate water deficit (W2: 55–70% θ_f_), mild water deficit (W3: 65–80% θ_f_), and full irrigation (W4: 75–90% θ_f_)—and four nitrogen application rates—no nitrogen (N0, 0 kg·ha^−1^), low nitrogen (N1, 80 kg·ha^−1^), medium nitrogen (N2, 160 kg·ha^−1^), and high nitrogen (N3, 240 kg·ha^−1^)—were established to systematically analyze the effects of water–nitrogen coupling on osmotic adjustment substances, yield, and forage quality of alfalfa (*Medicago sativa* L.) leaves. The results showed that: (1) Proline (Pro) content increased significantly with intensified water deficit, with W1 being 82.29% higher than W4 on average. Soluble protein (SP) and soluble sugar (SS) contents increased with increasing water availability, with their average values under W4 being 26.50% and 36.92% higher than those under W1, respectively. Increasing nitrogen application significantly improved the accumulation of osmotic adjustment substances, with Pro reaching the lowest value at N2, SP peaking at N2, and SS peaking at N3. (2) Yield increased significantly with higher irrigation, and increased first and then decreased with nitrogen application. Yield under W4 was 94.20% higher than under W1, and N2 increased yield by 12.45–50.65% compared with other nitrogen levels. (3) Under the W4N2 treatment, crude protein (CP) content and relative feed value (RFV) increased by 34.54% and 51.10%, respectively, compared with W1N0, while acid detergent fiber (ADF) and neutral detergent fiber (NDF) decreased by 28.74% and 24.44%, respectively. (4) Correlation analysis indicated that Pro content was significantly positively correlated with ADF and NDF but negatively correlated with yield, CP, and RFV. In contrast, SP and SS contents were significantly negatively correlated with ADF and NDF and positively correlated with yield, CP, and RFV. (5) Principal component analysis identified that the combination of full irrigation (W4: 75–90% θ_f_) and medium nitrogen application (N2, 160 kg·ha^−1^) optimizes both yield and forage quality by balancing osmotic adjustment substances.

## 1. Introduction

Globally, arid and semi-arid regions account for approximately one-third of the terrestrial area, while cultivable land suitable for stable agriculture represents less than 10% of the world’s land resources. Water scarcity has therefore become a major constraint on agricultural productivity [1]. China faces similar challenges, as nearly half of its territory is categorized as arid or semi-arid. Agricultural production and grassland ecosystems are widely restricted by insufficient water availability [2]. In addition, nitrogen deficiency commonly coexists with water scarcity on the Loess Plateau. Drought stress disrupts plant water balance, whereas appropriate nitrogen application helps improve nutrient supply and enhance drought tolerance [3]. Thus, nitrogen has become another key factor limiting crop productivity in this region after water. As an essential nutrient for plant growth, nitrogen plays a vital role in photosynthetic efficiency, biomass accumulation, and internal metabolic processes [4]. Therefore, exploring the physiological responses of typical forage species under varying water–nitrogen regimes will help elucidate water–nitrogen interaction mechanisms and provide theoretical support for sustainable agro-pastoral development in arid and semi-arid regions.

Alfalfa (*Medicago sativa* L.) is widely cultivated in water-limited regions due to its strong adaptability and high nutritional value. It is recognized as a crucial forage crop for enhancing grassland productivity and ecological function. However, in arid zones with insufficient water and nutrient supply, alfalfa production often suffers from decreased yield and forage quality [5]. Understanding the regulatory effects of water–nitrogen interactions on alfalfa physiological processes and yield-quality formation is therefore essential for developing efficient resource management strategies. Water and nitrogen are key factors affecting crop growth, development, and quality, acting both independently and interactively to modulate photosynthetic performance and biomass accumulation [6]. In arid and semi-arid environments, rational water–nitrogen management serves as an effective means to alleviate resource limitations and improve crop performance [7]. Appropriate water–nitrogen coordination not only enhances growth and photosynthetic capacity but also improves the soil microenvironment and facilitates root nutrient acquisition [8].

A substantial body of research has demonstrated that water–nitrogen coupling significantly influences crop yield and quality across different plant species, including cereals, vegetables, fruit trees, and perennial forage crops. For instance, Dong et al. [9] reported that optimal water–nitrogen regulation improved leaf expansion, photosynthetic performance, biomass production, and resource-use efficiency in wheat, enabling coordinated high yield and high efficiency in the piedmont plains of Hebei Province. Liu et al. [10] found that moderate water restriction increased soluble sugar and carotenoid contents in cherry tomatoes, thereby improving fruit quality, whereas excessive nitrogen application provided no yield benefit and increased the risk of nitrogen loss. Li et al. [11] showed that water–nitrogen interactions significantly affected photosynthetic efficiency and nut quality in macadamia trees in Yunnan, and that unsuitable moisture or excessive nitrogen inhibited photosynthesis and reduced kernel percentage. Wen et al. [12] reported that water–nitrogen coupling significantly affected biomass and forage quality of king grass, where appropriate water–nitrogen matching increased yield and crude protein content, while drought or excessive nitrogen adversely impacted physiological metabolism and quality formation. Collectively, these studies highlight the universal role of water–nitrogen interaction in regulating crop yield and quality, and emphasize that optimal matching of water and nitrogen is key to achieving high productivity and superior quality.

Osmotic adjustment substances serve as important physiological indicators of plant stress tolerance, and their variation reflects metabolic status under environmental stresses. The coordination between water and nitrogen markedly influences osmotic regulation and stress-responsive metabolism. Water deficit induces stomatal closure and limits CO_2_ assimilation, resulting in inhibited photosynthetic carbon fixation and excessive reactive oxygen species (ROS) accumulation, which leads to membrane lipid peroxidation and cellular damage [13]. Beyond water effects, nitrogen availability also influences the accumulation patterns of osmolytes. Moderate nitrogen supply enhances nitrate reductase (NR) and glutamine synthetase (GS) activities, promoting nitrogen assimilation and protein synthesis, thus increasing soluble protein (SP) content while preventing excessive proline (Pro) accumulation; whereas excessive nitrogen disrupts carbon–nitrogen balance and triggers an increase in soluble sugar (SS) and Pro overaccumulation [14]. Lou et al. [15] further suggested that the degree of coordination between water and nitrogen determines osmotic regulation capacity, where moderate water supply combined with medium nitrogen application promotes coordinated SP and SS accumulation, reduces membrane peroxidation products (MDA), and helps maintain higher photosynthetic activity and stress tolerance.

Despite extensive evidence on crop responses to water and nitrogen supply, most existing studies related to water–nitrogen regulation have primarily focused on individual factors or emphasized yield responses alone. Systematic investigations into the physiological mechanisms underlying the dynamic changes in osmotic adjustment substances across growth stages, and their quantitative relationships with the synergistic formation of yield and forage quality, remain limited, particularly for alfalfa under water–nitrogen interaction. Compared with many previously studied non-alfalfa crops, alfalfa is a perennial forage species characterized by repeated regrowth, sustained nitrogen fixation and assimilation capacity, and long-term exposure to fluctuating water–nitrogen conditions, which may result in distinct osmotic regulation strategies and yield–quality formation mechanisms.

Supported by National Natural Science Foundation projects, our research group has conducted long-term field trials (since 2017) on alfalfa water–nitrogen regulation at the Jingtai Irrigation Experimental Station in Gansu, China. These studies demonstrated that sufficient irrigation combined with moderate nitrogen input significantly improved alfalfa growth, yield, forage quality, and resource-use efficiency in the inland arid region of Northwest China [16,17], leading to the establishment of a regional adaptive water–nitrogen management strategy. However, the underlying physiological mechanisms responsible for this synergistic regulation remain unclear.

Therefore, this study employed a precisely controlled pot experiment under arid–semi-arid conditions to minimize environmental heterogeneity and systematically investigate the dynamic responses of osmotic adjustment substances (proline, soluble protein, and soluble sugar) in alfalfa across different growth stages, as well as their quantitative relationships with yield and forage quality traits (crude protein, acid detergent fiber, neutral detergent fiber, and relative feed value). This work aims to elucidate the physiological mechanisms underlying alfalfa adaptation to water–nitrogen coupling and to provide a theoretical basis for high-efficiency alfalfa production in arid and semi-arid regions of Northwest China.

## 2. Results

### 2.1. Responses of Osmotic Adjustment Substances to Water–Nitrogen Coupling

#### 2.1.1. Proline Content

**Figure 1:**
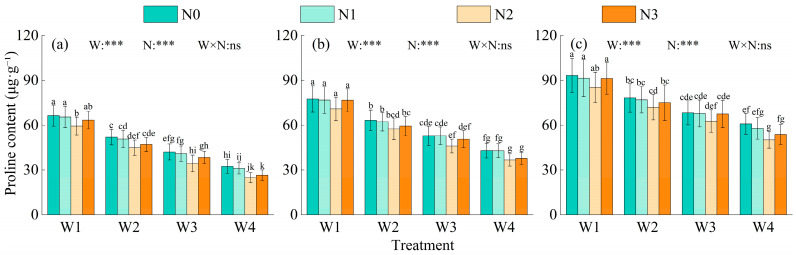
Effects of water–nitrogen coupling on proline content of Alfalfa. Note: Values are means ± standard deviation (STDEV.S, *n* = 3). Different lowercase letters indicate significant differences among treatments (*p* < 0.05, Duncan’s test). Growth stages: (**a**) branching stage, (**b**) budding stage, (**c**) initial flowering stage. Different colors represent different nitrogen levels. *** indicates significance at *p* < 0.001, while ns indicates no significant difference.

Proline content in alfalfa leaves at all growth stages was extremely significantly affected by irrigation level and nitrogen application (*p* < 0.001), whereas their interaction showed no significant effect (Figure 1). Proline content increased from branching stage to budding stage and further to flowering stage. Under the same nitrogen level, proline content followed the order W1 > W2 > W3 > W4. Compared with W1, W2, and W3, W4 decreased proline content by 42.55–49.05%, 29.56–35.95%, and 16.99–24.65%, respectively. Under the same irrigation level, proline content first decreased and then increased, with the lowest value at N2, which was 9.12–18.02%, 7.79–15.21%, and 1.03–7.04% lower than N0, N1, and N3, respectively. Among all treatments, W4N2 showed the lowest proline content, 5.27–52.91% lower than the other treatments.

#### 2.1.2. Soluble Protein

**Figure 2:**
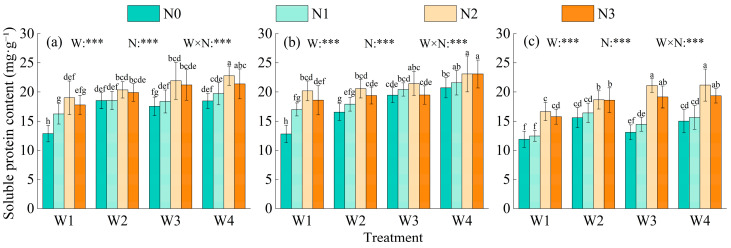
Effects of water–nitrogen coupling on soluble protein content of Alfalfa. Note: Values are means ± standard deviation (STDEV.S, *n* = 3). Different lowercase letters indicate significant differences among treatments (*p* < 0.05, Duncan’s test). Growth stages: (**a**) branching stage, (**b**) budding stage, (**c**) initial flowering stage. Different colors represent different nitrogen levels. *** indicates significance at *p* < 0.001.

Irrigation, nitrogen fertilization, and their interaction had extremely significant effects on soluble protein content (*p* < 0.001) (Figure 2). Soluble protein content in branching and budding stages was similar, with slightly higher values at the budding stage, while the flowering stage exhibited a distinct reduction. Under the same nitrogen level, soluble protein content generally increased as irrigation improved (W1 < W2 < W3 < W4). Compared with W1, W2, and W3, W4 increased soluble protein content by 20.08–43.98%, 6.86–12.54%, and 4.01–8.22%, respectively. A quadratic response to nitrogen fertilizer was observed, with the highest value at N2, which was 17.51–48.35%, 12.67–22.10%, and 2.96–7.77% higher than N0, N1, and N3, respectively. W4N2 exhibited the highest soluble protein content, 4.00–78.27% higher than the other treatments.

#### 2.1.3. Soluble Sugar

**Figure 3:**
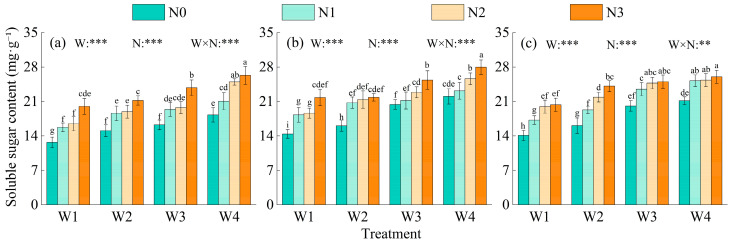
Effects of water–nitrogen coupling on soluble sugar content in alfalfa. Note: Values are means ± standard deviation (STDEV.S, *n* = 3). Different lowercase letters indicate significant differences among treatments (*p* < 0.05, Duncan’s test). Growth stages: (**a**) branching stage, (**b**) budding stage, (**c**) initial flowering stage. Different colors represent different nitrogen levels. ** and *** indicate significance at *p* < 0.001 and *p* < 0.001, respectively.

Soluble sugar (SS) content was extremely significantly affected by irrigation, nitrogen input, and their interaction (*p* < 0.01 or *p* < 0.001) (Figure 3). Among the growth stages, the budding stage showed slightly higher SS levels than the branching stage, while the flowering stage had the lowest SS content. Under the same nitrogen level, SS content increased with irrigation improvement (W1 < W2 < W3 < W4). Compared with W1, W2, and W3, W4 increased SS content by 29.20–49.13%, 18.47–30.18%, and 8.25–12.74%, respectively. SS content also increased steadily with nitrogen application (N0 < N1 < N2 < N3), and N3 increased SS by 30.65–50.80%, 14.59–21.24%, and 5.77–12.85% compared with N0, N1, and N2, respectively. Among all treatments, W4N3 had the highest SS content, which was 5.77–94.83% greater than other treatments.

### 2.2. Variation Characteristics of Yield

**Figure 4:**
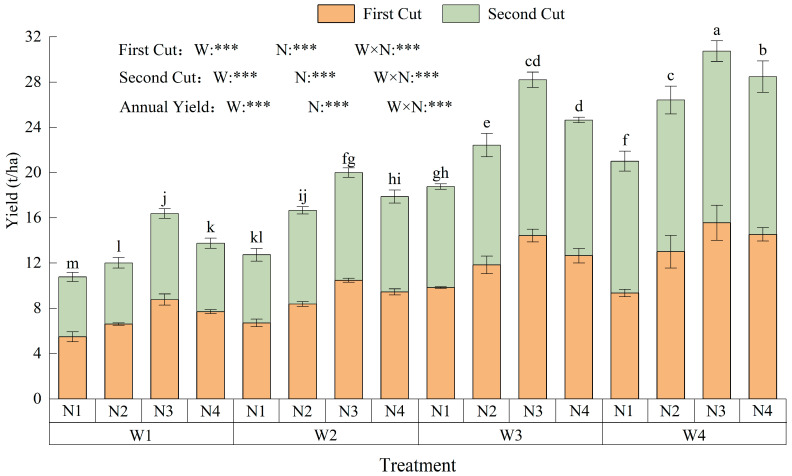
Alfalfa yield under different water and nitrogen treatments across two harvests. Note: Bars represent the dry matter yields of the first and second cuttings, stacked to show the total annual yield. Error bars indicate standard deviation (STDEV.S, *n* = 3). Different lowercase letters indicate significant differences in total annual yield among treatments at the 0.05 level (Duncan’s test). *** indicates significance at *p* < 0.001.

Irrigation, nitrogen application, and their interaction all had extremely significant impacts on alfalfa yield (*p* < 0.001) (Figure 4). The first-cut average yield was 10.30 t·ha^−1^, slightly higher than that of the second cut. Annual total yield increased continuously with irrigation level. Under the same nitrogen level, yield followed the order W1 < W2 < W3 < W4, and W4 increased the yield by 87.83–119.83%, 53.70–64.99%, and 9.01–17.79% compared with W1, W2, and W3, respectively. Nitrogen level showed a unimodal response, with N2 achieving the highest yield; compared with N0, N1, and N3, N2 increased yield by 46.38–57.18%, 16.37–36.29%, and 7.93–19.09%, respectively. Among all treatments, W4N2 achieved the highest annual yield, 7.94–185.39% higher than the other treatments.

### 2.3. Responses of Forage Quality Attributes

**Figure 5:**
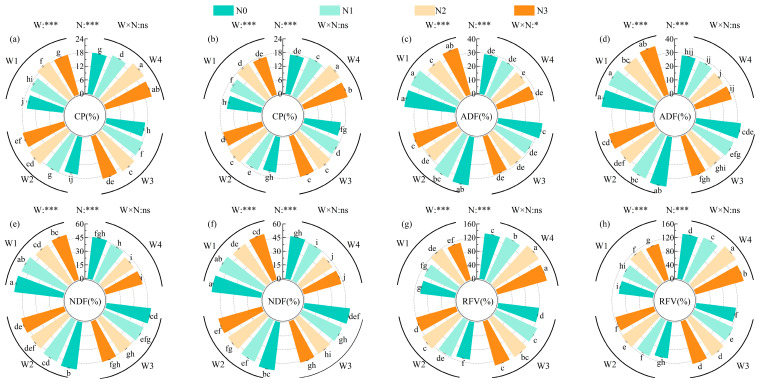
Effects of water–nitrogen coupling on crude protein (CP), acid detergent fiber (ADF), neutral detergent fiber (NDF) contents, and relative feed value (RFV) of alfalfa. Note: (**a**,**b**) Crude protein (CP); (**c**,**d**) acid detergent fiber (ADF); (**e**,**f**) neutral detergent fiber (NDF); (**g**,**h**) relative feed value (RFV). Panels (**a**,**c**,**e**,**g**) represent data from the first cut, while panels (**b**,**d**,**f**,**h**) represent data from the second cut. Values are means ± standard deviation (STDEV.S, *n* = 3). Different lowercase letters indicate significant differences among treatments (*p* < 0.05, Duncan’s test). Different colors represent different nitrogen levels. * and *** indicate significance at *p* < 0.05, and *p* < 0.001, respectively; ns indicates no significant difference.

Irrigation and nitrogen levels had extremely significant effects on crude protein (CP), acid detergent fiber (ADF), neutral detergent fiber (NDF), and relative feed value (RFV) (*p* < 0.001), while their interaction showed significance only for ADF in the first cut; no significant interaction was detected for the other indices in either cut (Figure 5a–h). The mean CP and RFV in the first cut were 4.79% and 2.82% higher than in the second cut, while ADF and NDF means were 2.38% and 1.82% lower, respectively. Under the same irrigation level, CP and RFV exhibited unimodal responses to nitrogen (maximum at N2), while ADF and NDF showed a U-shaped trend (minimum at N2). Under the same nitrogen level, CP and RFV increased from W1 to W4, whereas ADF and NDF displayed the opposite pattern. The optimal forage quality was observed in W4N2, where CP and RFV reached 21.06% and 153.53, while ADF and NDF were 26.83% and 41.21%, respectively (*p* < 0.001).

### 2.4. Correlations Among Osmotic Adjustment Substances, Yield, and Forage Quality

**Figure 6:**
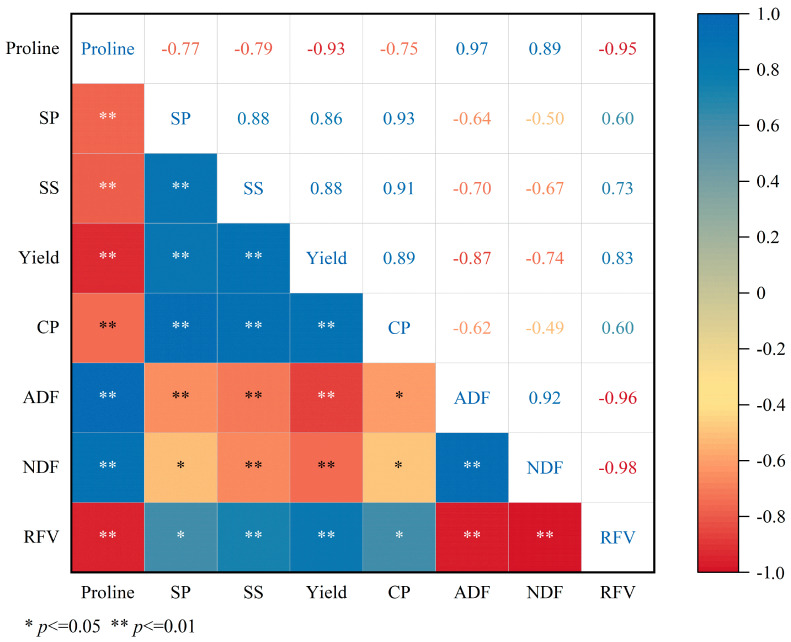
Correlation analysis among osmotic adjustment substances, yield, and forage quality traits of alfalfa under water–nitrogen coupling.

Pearson correlation analysis (Figure 6) revealed multiple significant correlations among osmotic adjustment substances (Pro, SP, SS), yield, and forage quality parameters (CP, ADF, NDF, and RFV). Most correlation coefficients exceeded 0.60 or were below −0.60, indicating strong positive or negative linear relationships. Pro was strongly positively correlated with ADF (r = 0.97) and NDF (r = 0.89), and negatively correlated with all other indices. SP and SS were significantly positively correlated with RFV (r = 0.60 and 0.73), while negatively correlated with ADF (r = −0.64 and −0.70) and NDF (r = −0.50 and −0.67). Yield had the strongest positive correlation with CP (r = 0.94), and showed significant negative correlations with ADF (r = −0.87) and NDF (r = −0.74).

### 2.5. Principal Component Analysis

**Figure 7:**
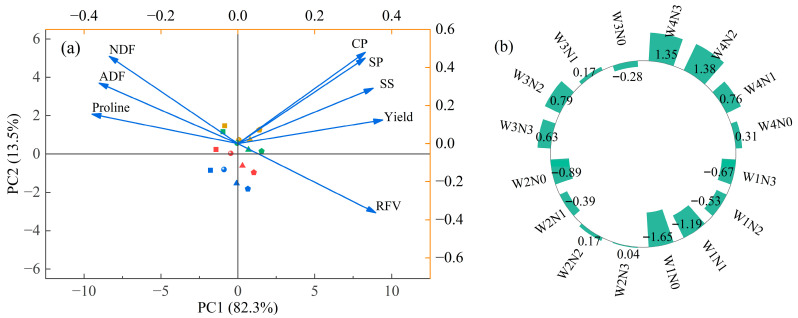
Principal component loadings (**a**) and comprehensive scores of treatments (**b**). Note: (**a**) PCA biplot showing the factor loadings of alfalfa’s osmotic adjustment substances (proline, soluble protein [SP], soluble sugar [SS]), forage quality traits (crude protein [CP], acid detergent fiber [ADF], neutral detergent fiber [NDF], relative feed value [RFV]), and dry matter yield (DMY) under water–nitrogen coupling. PC1 and PC2 explained 82.3% and 13.5% of the total variance, respectively. (**b**) Comprehensive PCA scores of different treatments calculated from PC1 and PC2. Blue, red, yellow, and green represent nitrogen levels N0, N1, N2, and N3, respectively; squares, circles, triangles, and pentagons correspond to water levels W1, W2, W3, and W4, respectively.

Principal component analysis (Figure 7a) showed that PC1 and PC2 explained 82.3% and 13.5% of the total variance, respectively, with a cumulative contribution of 95.8%. PC1 was the dominant component. CP, SP, SS, yield, and RFV had high positive loadings on PC1, whereas Pro, ADF, and NDF exhibited strong negative loadings, indicating opposite variation patterns among these traits. PC1 comprehensively represented the dominant direction of yield–quality variation, with positive loadings reflecting “high yield–high quality” characteristics and negative loadings indicating “stress response–fiber accumulation”. The integrated treatment performance (Figure 7b) demonstrated that W4 achieved the highest scores among irrigation levels, N2 scored highest among nitrogen levels, and W4N2 achieved the highest overall score, indicating the most favorable water–nitrogen management strategy under the current experimental conditions.

## 3. Discussion

### 3.1. Effects of Water–Nitrogen Regulation on Osmotic Adjustment Substances

Osmotic adjustment substances play a crucial physiological role in regulating cellular osmotic potential, stabilizing cellular structures, and supporting carbon–nitrogen metabolism during plant development and stress adaptation [18]. In response to water conditions, alfalfa exhibited significant changes in osmotic adjustment metabolites. In this study, Pro content increased with enhanced drought stress, whereas SP and SS levels increased significantly with higher irrigation supply. In contrast to the present results, Zhou et al. [19] in spring wheat and Ding et al. [20] in winter rapeseed reported that Pro, SP, and SS all increased under intensified drought stress, with a slight decrease only under extreme water deficit. These discrepancies may be attributed to differences in species traits (perennial legume versus annual crops), as well as variations in climatic conditions and experimental settings. Accumulation of osmolytes is widely recognized as a common drought resistance strategy, lowering cellular osmotic potential, maintaining water uptake, and protecting membrane stability [21]. Consistent with our results, Chai et al. [22] found in pomegranate seedlings that sufficient water supply maintains photosynthetic carbon availability, thereby supporting protein and sugar biosynthesis, enhancing metabolic homeostasis. These findings suggest that osmotic regulation in alfalfa under varying water conditions relies more on sustaining metabolic activity rather than solely on stress-induced accumulation.

Under nitrogen regulation, osmotic adjustment substances in alfalfa displayed differentiated responses. In this study, Pro content was lowest at N2, while SP increased with nitrogen input before decreasing at high levels, peaking at N2. SS continuously increased with nitrogen input and reached the maximum at N3. These results agree with findings in *Phoebe zhennan* seedlings [23], indicating that moderate nitrogen maintains carbon–nitrogen balance, whereas excessive nitrogen disrupts this coordination, leading to metabolic imbalance characterized by re-elevated Pro and decreased SP. Similarly, Liu et al. [24] showed that appropriate nitrogen supply in maize suppressed stress-induced Pro accumulation while increasing soluble protein and sugar levels. Huang et al. [25] found in rice that proper nitrogen management inhibited excessive Pro accumulation and promoted synthesis and translocation of soluble sugar and proteins. These outcomes may be attributed to enhanced activities of key enzymes such as NR and GS under moderate nitrogen supply, promoting nitrate reduction and assimilation and improving nitrogen metabolic balance and physiological status, thereby reducing stress responses and stimulating protein biosynthesis [26]. Excessive nitrogen, however, may lead to a mismatch between nitrogen assimilation and carbon partitioning, increasing soluble sugar accumulation while secondarily inducing Pro re-accumulation, resulting in a metabolic pattern defined as “increased sugars, decreased proteins, and rebound Pro” [27].

In addition to treatment effects, the variation in osmotic adjustment substances across different growth stages reflects shifts in physiological demand and carbon allocation in alfalfa. During the branching and budding stages, vegetative growth predominates, and sufficient water supply combined with moderate nitrogen availability promotes photosynthetic carbon assimilation and nitrogen metabolism, resulting in coordinated accumulation of soluble protein and soluble sugar. In contrast, at the initial flowering stage, increased carbon consumption by reproductive organ development may partially reduce soluble sugar levels, particularly under water-deficit conditions. However, under the W4N2 treatment, adequate water availability and moderate nitrogen input likely maintained higher photosynthetic activity and metabolic balance, thereby alleviating carbon depletion and sustaining relatively stable osmotic regulation.

Existing literature suggests that alfalfa tends to regulate osmotic adjustment through active metabolic maintenance rather than passive stress-induced accumulation, owing to its strong photosynthetic capacity and efficient nitrogen assimilation. In this study, proline content increased mainly under intensified water stress, whereas soluble protein and soluble sugar accumulated predominantly under conditions of sufficient water supply and appropriate nitrogen input. Overall, water availability played a dominant role in determining stress intensity in alfalfa, as reflected by proline accumulation, whereas nitrogen primarily regulated metabolic adjustment by modulating soluble protein and soluble sugar accumulation under different water conditions. These physiological responses provide mechanistic support for our previous field observations that the “sufficient irrigation + moderate nitrogen” management mode performs optimally in Northwest China.

### 3.2. Effects of Water–Nitrogen Regulation on Yield and Forage Quality

As a key indicator of grassland ecosystem productivity, alfalfa yield reflects crop adaptability and water–nutrient use efficiency [28]. Forage quality, determined by CP, ADF, NDF, and RFV, directly affects nutritive value and digestibility [29]. Irrigation and nitrogen serve as two major management factors affecting biomass accumulation and forage quality formation [18,21,30]. Thus, understanding the response patterns of yield and quality to water–nitrogen treatments is critical for improving production efficiency and forage value of alfalfa.

In this study, alfalfa yield significantly increased with irrigation supply, with W4 improving annual yield by 87.80–119.88% relative to W1. Nitrogen showed a typical unimodal response, with the highest yield at N2 and a decline at N3. Similar observations were reported in other crops: Li et al. [31] found that yield of adzuki bean under normal irrigation increased by 84.50–198.70% compared with drought conditions; Liu et al. [24] noted that maize yield and water-use efficiency peaked at 200 kg·ha^−1^ nitrogen, while higher input produced no further yield benefit. The enhancement in yield with irrigation may result from improved stomatal conductance, electron transport efficiency, CO_2_ assimilation, and stable canopy structure [32,33]. Moderate nitrogen enhances activities of key nitrogen metabolic enzymes (NR, GS), promoting protein synthesis and photosynthetic carbon assimilation [34], whereas excessive nitrogen disrupts carbon–nitrogen balance and lowers nutrient-use efficiency, suppressing yield gains [35].

Regarding forage quality, CP and RFV increased with irrigation improvement, while ADF and NDF declined, and the best quality was observed under N2. Ma et al. [36] reported that high water availability in Ningxia increased CP and reduced fiber components in alfalfa, improving RFV. A meta-analysis by Yin et al. [32] indicated that moderate nitrogen typically increases CP by 7.30% and reduces ADF and NDF by 5.60% and 3.00%, while over-fertilization weakens these benefits. These responses may be attributed to sufficient irrigation enhancing nitrogen absorption and assimilation, suppressing drought-induced lignin deposition, thus improving nutritive value. Moderate nitrogen supports balanced metabolic allocation toward protein synthesis, whereas excessive nitrogen promotes structural carbohydrate deposition and stem proportion, resembling drought-like effects that elevate fiber contents and reduce digestibility [29,37].

Collectively, irrigation served as the decisive factor determining the yield and forage quality potential of alfalfa, whereas appropriate nitrogen supply further optimized both outcomes. Water availability established the upper boundary of biomass accumulation and forage quality formation, while nitrogen input fine-tuned carbon–nitrogen allocation and quality traits within this boundary. Under adequate irrigation, moderate nitrogen input significantly enhanced both yield and forage nutritive value, explaining why the W4N2 treatment simultaneously achieved the highest yield and optimal quality in this study.

### 3.3. Mediating Role of Osmotic Adjustment Substances in Yield and Quality Formation

Osmotic adjustment substances play a crucial intermediary role in determining yield and forage quality in alfalfa [38]. Correlation and PCA analyses in this study revealed that Pro was strongly and positively associated with ADF and NDF but negatively correlated with yield, CP, and RFV. In contrast, SP and SS exhibited positive correlations with yield, CP, and RFV. These results indicate that osmotic metabolites not only reflect plant stress responses but also influence carbon–nitrogen metabolism and structural deposition, thereby affecting yield–quality performance.

Excessive Pro accumulation was closely associated with reduced yield, likely due to the metabolic cost required for Pro synthesis, which limits allocation of carbon and nitrogen skeletons toward productive metabolism and inhibits biomass formation [39]. Conversely, increased SP and SS were associated with higher yields. SP elevation indicates enhanced nitrogen assimilation and protein metabolism, while SS accumulation reflects abundant photosynthetic carbon supply supporting energy metabolism and biomass production [24].

The superiority of the W4N2 treatment revealed by PCA can be explained by its integrated regulation of carbon and nitrogen metabolism. Adequate water availability maintains photosynthetic capacity and carbon supply, while moderate nitrogen input enhances nitrogen assimilation efficiency, which is commonly reflected by the activity of key nitrogen metabolic enzymes such as nitrate reductase (NR) and glutamine synthetase (GS), as widely reported in previous studies, thereby promoting soluble protein accumulation and sustaining soluble sugar availability for energy metabolism. At the same time, the balanced water–nitrogen status reduces excessive proline accumulation, lowering metabolic costs associated with stress defense. This coordinated physiological regulation provides a mechanistic link between osmotic adjustment, biomass production, and forage quality formation, explaining why W4N2 simultaneously achieved high yield and superior quality.

Similarly, the increase in Pro coincided with elevated ADF and NDF, suggesting enhanced deposition of structural carbon and reduced digestibility. Meanwhile, increases in SP and SS were associated with higher CP and RFV, implying synergistic support for protein accumulation and energy metabolism. Comparable findings have been reported in other crops, such as pineapple [40] and king grass [12], where suppression of Pro and enhancement of SP and SS under suitable water–nitrogen coordination improved quality traits.

This study demonstrated that reduced water–nitrogen stress conditions promoted SP and SS, which were strongly associated with higher yield and improved forage quality, whereas excessive Pro accumulation was linked to increased fiber content and reduced digestibility. In the regulatory pathway identified, water availability primarily determined overall carbon supply through its control of photosynthetic activity, while nitrogen availability governed nitrogen assimilation efficiency and protein synthesis. Osmotic adjustment substances therefore functioned as key physiological mediators linking water–nitrogen regulation with carbon–nitrogen metabolism, biomass production, and forage quality formation. Overall, osmotic adjustment substances responded more sensitively to short-term water–nitrogen regulation, yield reflected the integrated outcome of carbon assimilation and biomass accumulation, whereas forage quality traits showed a comparatively buffered but directional response driven by structural carbon–nitrogen allocation. Differences from some field-based studies may further reflect the controlled pot conditions and the precise water–nitrogen regulation applied in this experiment, which reduced environmental heterogeneity compared with open-field systems. These findings not only provide a physiological explanation for the superiority of the “sufficient irrigation + moderate nitrogen” strategy but also offer a practical reference for optimizing water–nitrogen management in alfalfa production in semi-arid regions.

It should be noted that this study was conducted under controlled pot conditions, which may differ from field environments in terms of root growth space, soil aeration, and water–nutrient dynamics. Therefore, caution is required when extrapolating these results directly to field-scale production systems. Further field experiments under different soil types and precipitation regimes are needed to validate the applicability of the proposed water–nitrogen management strategy.

## 4. Materials and Methods

### 4.1. Experimental Site and Plant Materials

The experiment was conducted from May to October 2024 at the experimental farm of the College of Grassland Science, Gansu Agricultural University, Lanzhou, China (103°40′ E, 36°03′ N). The site is characterized by a temperate semi-arid continental climate, with an average altitude of 1525 m, mean annual temperature of 10.3 °C, frost-free period of approximately 180 days, and annual precipitation of 400–600 mm, mainly occurring from July to September. The mean annual potential evaporation is about 1410 mm, and annual sunshine duration ranges from 2100 to 2600 h.

The tested alfalfa cultivar was ‘Gannong No. 3’, provided by the College of Grassland Science, Gansu Agricultural University. Plants were grown in resin pots 20 cm in height and 20 cm in diameter, with an effective volume of approximately 6.3 L, each filled with about 6.5 kg of substrate. The experimental substrate consisted of a 5:1 (*w*/*w*) mixture of surface loessial soil collected from the experimental field and a commercial seedling substrate (Shenxian Lüyuan Seedling Substrate Co., Ltd., Liaocheng, Shandong, China). The loessial soil provided a representative local soil background, while the addition of a small proportion of commercial seedling substrate was intended to improve the physical structure and aeration of the potting medium, thereby facilitating uniform seedling establishment and better simulating local field soil conditions under controlled pot conditions. The physicochemical properties of the mixed substrate, including pH, field capacity, bulk density, organic matter content, total nitrogen, available nitrogen, available phosphorus, and available potassium, were determined before sowing and are summarized in Table 1.

Urea (N ≥ 46%) was used as the sole nitrogen source. Phosphorus fertilizer was applied as superphosphate at 40.0 kg·ha^−1^ (16% P_2_O_5_), and potassium fertilizer as potassium sulfate at 19.2 kg·ha^−1^ (50% K_2_O). All fertilizers were applied twice, once before regrowth of the first cut and once before regrowth of the second cut. Other field management practices followed the local standard production protocol for alfalfa.

### 4.2. Experimental Design and Field Management

A completely randomized block design was adopted in a rain-shelter facility without temperature control, with open sides for ventilation and a plastic film roof to exclude rainfall. The pots were buried to approximately two-thirds of their height in the soil to simulate field-like conditions under different water–nitrogen combinations. Four irrigation levels were established based on 85% of field capacity (27.30% volumetric water content), defined as θ_f_: 45–65% θ_f_ (W1, severe water deficit), 55–70% θ_f_ (W2, moderate deficit), 65–80% θ_f_ (W3, mild deficit), 75–90% θ_f_ (W4, full irrigation). Four nitrogen levels were set at 0 (N0), 80 (N1), 160 (N2), and 240 (N3) kg·ha^−1^. The combinations of irrigation and nitrogen levels resulted in 16 treatments, each with three replicates, for a total of 48 pots [41]. The detailed water and nitrogen gradients are shown in Table 2.

Before sowing (14 May 2024), the experimental area was ploughed, levelled, and weeded, and all pots were buried in the soil and numbered. No herbicides or insecticides were used during the experiment, and weeds and pests were controlled manually. Fertilization followed a “basal + topdressing” scheme, with 40% of the total fertilizer applied as basal fertilizer before sowing and the remaining 60% applied as topdressing in two equal splits (30% each) at the branching stage of the first and second cuts.

Soil volumetric water content (VWC) was monitored every 3 days using a portable time-domain reflectometer (TRIME-PICO-IPH, IMKO, Ettlingen, Germany) at 0–20 cm depth, with increased monitoring frequency during hot periods. When the measured VWC of a pot fell below the lower threshold of its assigned treatment, irrigation was applied manually to restore soil moisture to the upper limit of the target range. The amount of irrigation water added to each pot was calculated according to the difference between the measured VWC and the target upper limit, combined with the effective soil volume of the pot, and water was applied evenly to ensure uniform wetting. Specifically, irrigation amount for each pot was determined as the product of the difference between target and measured volumetric water content and the effective soil volume of the pot, ensuring precise restoration of soil moisture to the designated treatment range. To ensure measurement accuracy and water control consistency, soil water status was additionally verified every 7 days using the gravimetric oven-drying method.

### 4.3. Measurements and Analytical Methods

#### 4.3.1. Osmotic Adjustment Substances

In both the first and second growth cycles (cuts), fully expanded mature leaves of alfalfa were sampled at the branching, budding, and initial flowering stages for determination of osmotic adjustment substances. For each treatment, three plants were randomly selected for sampling. Leaf samples were immediately placed in iceboxes, transported to the laboratory at low temperature, and promptly extracted and analyzed to ensure data reliability. For statistical analysis, the mean values of the two cuts at each growth stage were used. Proline (Pro) content (μg·g^−1^ FW) was determined using the acid ninhydrin method. Soluble protein (SP) content (mg·g^−1^ FW) was measured by the Coomassie Brilliant Blue G-250 method. Soluble sugar (SS) content (mg·g^−1^ FW) was determined using the anthrone colorimetric method.

#### 4.3.2. Yield Determination

Aboveground yield of alfalfa was determined at the initial flowering stage of both growth cycles (first and second cuts). Before harvest, plants with uniform growth were selected within each treatment, and shoots were cut at 5 cm above the soil surface. After removal of weeds and dead tissue, fresh weight was recorded immediately. The samples were first dried at 105 °C for 1 h and then oven-dried at 75 °C to constant weight to obtain dry matter. Aboveground yield (t·ha^−1^) was calculated from dry weight based on the surface area represented by each pot.

#### 4.3.3. Forage Quality Measurements

Forage quality traits were measured at the initial flowering stage of both cuts. The dry plant materials obtained in Section 4.3.2 were ground to pass a 1 mm sieve and used for determination of *CP*, *NDF*, and *ADF*.

(1) Crude protein (CP)

Total nitrogen content was determined by the Kjeldahl method [32], and *CP* was calculated as:

(1) $$CP\left(\%\right)=TN\left(\%\right)\times 6.25$$

(2) Neutral detergent fiber (NDF)

*NDF* content was determined according to the Van Soest method [32]. *NDF* was calculated as:

(2) $$NDF\left(\%\right)=\frac{W_{2}-W_{1}}{S}$$

where $W_{2}$ is the mass of the filter bag plus residue after neutral detergent extraction and drying (g), $W_{1}$ is the mass of the empty filter bag (g), and S is the original sample mass (g).

(3) Acid detergent fiber (ADF)

*ADF* content was determined using the same procedure as for *NDF*, with acid detergent solution [17]. *ADF* was calculated as:

(3) $$ADF\left(\%\right)=\frac{W_{2}-W_{1}}{S}$$

(4) Relative feed value (RFV).

*RFV* was calculated based on *ADF* and *NDF* to comprehensively evaluate forage digestibility and intake potential, using the following empirical equations:

(4) $$DDM\left(\%\right)=88.9-\left[0.779\times ADF\left(\%\right)\right]$$

(5) $$DMI\left(\%\;of\;BW\right)=\frac{120}{NDF\left(\%\right)}$$

(6) $$RFV=\frac{DDM\times DMI}{1.29}$$

where *DDM* is digestible dry matter and *DMI* is dry matter intake as a percentage of body weight.

### 4.4. Data Analysis

All data were organized using Microsoft Excel 2021. Two-way analysis of variance (ANOVA) was performed using IBM SPSS Statistics 27.0 to evaluate the main effects of irrigation, nitrogen, and their interaction. When significant effects were detected, Duncan’s multiple range test was applied for mean separation at *p* < 0.05. One-way ANOVA was additionally used for comparisons within individual irrigation or nitrogen levels when appropriate. Principal component analysis (PCA) was conducted to explore the relationships among osmotic adjustment substances, yield, and forage quality traits under different water–nitrogen treatments. Figures were generated using Origin 2024b.

## 5. Conclusions

(1) Under drought conditions, Pro content markedly increased, whereas sufficient water supply significantly enhanced SP and SS accumulation. Regarding nitrogen response, Pro reached the lowest level at N2, SP peaked at N2, and SS continuously increased with nitrogen input, indicating a differentiated regulation pattern.
(2) Alfalfa yield consistently increased with higher irrigation levels, and exhibited a unimodal response to nitrogen, with the highest yield at the moderate nitrogen level (160 kg·ha^−1^). Forage quality was also improved under sufficient irrigation combined with moderate nitrogen, as reflected by increased CP and RFV and decreased ADF and NDF.
(3) Correlation and PCA analyses revealed that Pro was significantly positively correlated with ADF and NDF but negatively correlated with yield, CP, and RFV. In contrast, SP and SS showed significant positive correlations with yield, CP, and RFV.

Overall, the treatment combining high water supply and moderate nitrogen input (W4N2) achieved the highest performance in both yield and forage quality, consistent with our long-term field experiments. This water–nitrogen strategy is suitable for high-yield and high-quality alfalfa production in arid and semi-arid regions of Northwest China.

## Figures and Tables

**Table 1:** Physicochemical properties of the experimental substrate (0–30 cm).

Property	Field Capacity (cm^3^/cm^3^)	Bulk Density (g/cm^3^)	pH	Organic Matter (g/kg)	Total N (g/kg)	Available N (mg/kg)	Available P (mg/kg)	Available K (g/kg)
Value	27.3	0.94	7.7	16.44	0.27	68.55	3.71	0.24

**Table 2:** Experimental design of water and nitrogen treatments for alfalfa.

Treatment	Irrigation Level (% θ_f_)		Nitrogen Level (kg·ha^−1^)
W1N0	Severe water deficit	45–65%	0
W1N1		45–65%	80
W1N2		45–65%	160
W1N3		45–65%	240
W2N0	Moderate water deficit	55–70%	0
W2N1		55–70%	80
W2N2		55–70%	160
W2N3		55–70%	240
W3N0	Mild water deficit	65–80%	0
W3N1		65–80%	80
W3N2		65–80%	160
W3N3		65–80%	240
W4N0	Full irrigation	75–90%	0
W4N1		75–90%	80
W4N2		75–90%	160
W4N3		75–90%	240

## Data Availability

All data are incorporated into the article.

## References

[1] FAO (2021). The State of the World’s Land and Water Resources for Food and Agriculture—Systems at Breaking Point (SOLAW 2021).

[2] Li J. (2025). Driving Mechanism and Zoning Management of Ecosystem Service Supply and Demand Relationships in the Northwest Agro-Pastoral Ecotone. Doctoral Dissertation.

[3] Zeng Y., Mao J., Chen X., Xu X., Liang J., Liu Y. (2025). Osmotic regulation mechanisms of *Pinus tabuliformis* seedlings under the joint effects of nitrogen addition and drought stress. Sci. Silvae Sin..

[4] Li H., Chang W.Y. (2021). Exploring optimal film mulching to enhance potato yield in China: A meta-analysis. Agron. J..

[5] Wang C. (2024). Effects of Water and Nitrogen Regulation on Physiological Growth, Yield and Quality of *Lycium barbarum* || Alfalfa System. Master’s Thesis.

[6] Li J., Li Z., Zhang F., Fang D., Wang H. (2016). Effects of water and nitrogen supply on chlorophyll content and photosynthetic rate of greenhouse cucumber. Agric. Res. Arid Areas.

[7] Li J., Xiao Y., Wang X., Yang T., Zhang X., Li H., Yang W. (2025). Regulation effects of different water and nitrogen combinations on watermelon growth, yield and water and nitrogen use efficiency in sand removal land. Agric. Res. Arid Areas.

[8] Wang Z., Wang H., Wang J., Xu H., Liu S., Yao X. (2025). Effects of water and nitrogen interaction on root morphology and soil enzyme activity in drip-irrigated spring wheat in the Yanqi Basin. Agric. Res. Arid Areas.

[9] Dong W., Zhang Y. (2022). Effects of water–nitrogen interaction on physiological parameters and yield formation of different wheat varieties. Crops.

[10] Liu Y., Xia H., Li Y., Ren N. (2024). Effects of irrigation and nitrogen coupling on growth, yield and quality of cherry tomato under greenhouse cultivation during the whole growth period. J. Chin. Agric. Mech..

[11] Li S., He X., Yang L., Geng S., Ma J., Wang C. (2025). Effects of water–nitrogen interaction on photosynthetic physiology and fruit quality of macadamia. South China Fruits.

[12] Wen C. (2024). Effects of water and nitrogen coupling on yield and quality of king grass. J. Anhui Agric. Sci..

[13] He J., Chang C., Qin L., Lai C.H. (2023). Impacts of deficit irrigation on photosynthetic performance, productivity and nutritional quality of aeroponically grown Tuscan kale (*Brassica oleracea* L.) in a tropical greenhouse. Int. J. Mol. Sci..

[14] Ru C., Wang K., Hu X., Chen D., Wang W., Yang H. (2023). Nitrogen modulates the effects of heat, drought, and combined stresses on photosynthesis, antioxidant capacity, cell osmoregulation, and grain yield in winter wheat. J. Plant Growth Regul..

[15] Lou H., Zheng X., Wang P., Zhang M., Wang Y., Hu X., Mu Y., Huang J., Ai S., Zhang L. (2024). Effects of water and nitrogen coupling on osmotic regulators and stress resistance physiological characteristics of flue-cured tobacco in Southern Shaanxi. J. Hunan Agric. Univ. (Nat. Sci.).

[16] Wang C., Qi G., Ma Y., Yin M., Wang J., Kang Y., Jia Q., Gao Y., Tian R., Zhang R. (2024). Effects of water and nitrogen control on the growth physiology, yields, and economic benefits of *Lycium barbarum* plants in a *Lycium barbarum* + alfalfa system. Plants.

[17] Ma Y., Yu W., Chang W., Wang Y., Yin M., Kang Y., Qi G., Wang J., Zhao Y., Wang J. (2024). Effects of water and nitrogen regulation on soil environment and crop growth in a *Lycium barbarum*–alfalfa system. Plants.

[18] Zeng H. (2007). Effects of Soil Water and Nitrogen Fertilizer on Some Physiological Characters, Yield and Quality of Pepper. Master’s Thesis.

[19] Zhou P., Chen Z., Zhuang L., Xu H., Mu P., Li Y. (2013). Effects of irrigation and nitrogen interaction on osmoregulation substances content and yield of drip irrigation wheat. J. Shihezi Univ. (Nat. Sci.).

[20] Ding X. (2025). Effects of Water and Nitrogen Regulation on Physiological Characteristics, Yield and Quality of Drip-Fertigated Winter Oilseed Rape. Master’s Thesis.

[21] Zhang Z., Pei H., Li L., Jing M., Qin C., He Z., Zhang Z., Shen J., Lian H. (2025). Physiological responses of mung bean seedlings to nitrogen fertilizer under different water conditions. J. Jiangsu Agric. Sci..

[22] Chai Y., Guan S., Cui H., Xu J., Zhu X., Diao M., Kong Q. (2022). Effects of water and nitrogen interaction on photosynthetic fluorescence and physiological characteristics of pomegranate seedlings. J. Fruit Sci..

[23] Wang X., Hu H., Hu T., Zhang C., Wang X., Liu D. (2018). Effects of drought stress on osmotic adjustment and active oxygen metabolism in *Phoebe zhennan* seedlings and its alleviation by nitrogen application. Chin. J. Plant Ecol..

[24] Liu Y., Wang L., Li L., Xie J., Yue K., Wang J. (2022). Physiological mechanisms of nitrogen fertilization promoting yield and water use efficiency of dryland maize fields with plastic mulching. J. Yunnan Agric. Univ. (Nat. Sci.).

[25] Huang H., Shen T., Zhong L., Du J., Fu J., Zhou D., He H., Chen X. (2021). Effects of nitrogen application on yield and physiology of late rice under drought condition. Acta Agric. Boreali-Sin..

[26] Chen Z., Cao X., Niu J. (2021). Effects of melatonin on morphological characteristics, mineral nutrition, nitrogen metabolism, and energy status in alfalfa under high-nitrate stress. Front. Plant Sci..

[27] Wang J.W., Qi D.L. (2020). Irrigation and nitrogen supply methods: Effect on leaf physiological characteristics and yield of maize. Chin. Agric. Sci. Bull..

[28] Lv Y., Wang J., Yin M., Kang Y., Ma Y., Jia Q., Qi G., Jiang Y., Lu Q., Chen X. (2023). Effects of planting and nitrogen application patterns on alfalfa yield, quality, water–nitrogen use efficiency, and economic benefits in the Yellow River irrigation region of Gansu Province, China. Water.

[29] Huo W., Zhang Y., Zhang L., Shen C., Chen L., Liu Q., Zhang S., Wang C., Guo G. (2022). Effect of lactobacilli inoculation on protein and carbohydrate fractions, ensiling characteristics and bacterial community of alfalfa silage. Front. Microbiol..

[30] Feng H. (2025). Effects of Drip Irrigation Amount and Nitrogen Rate on Photosynthetic Characteristics and Fruit Yield Quality of Sunlight Greenhouse Tomatoes. Master’s Thesis.

[31] Li X., Zhang Y., Wang D., Luo H., Liu L., Wang J. (2015). Effects of coupling water and nitrogen on root physio-ecological indices and yield of adzuki bean. Chin. J. Eco-Agric..

[32] Yin M., Jiang Y., Ling Y., Ma Y., Qi G., Kang Y., Wang Y., Lu Q., Shang Y., Fan X. (2025). Optimizing lucerne productivity and resource efficiency in China’s Yellow River irrigated region: Synergistic effects of ridge-film mulching and controlled-release nitrogen fertilization. Agriculture.

[33] Kamran M., Yan Z., Jia Q., Chang S., Ahmad I., Ghani M.U., Hou F. (2022). Irrigation and nitrogen fertilization influence on alfalfa yield, nutritive value, and resource use efficiency in an arid environment. Field Crops Res..

[34] Du J., Shen T., Xiong Q., Zhu C., Peng X., He X., Fu J., Ouyang L., Bian J., Hu L. (2020). Combined proteomics, metabolomics and physiological analyses of rice growth and grain yield with heavy nitrogen application before and after drought. BMC Plant Biol..

[35] Zhang Y., Yu S., Li Z., Chang T., Xu Q., Xu H., Zhang J. (2022). Effects of excessive nitrogen fertilizer and soil moisture deficiency on antioxidant enzyme system and osmotic adjustment in tomato seedlings. Int. J. Agric. Biol. Eng..

[36] Ma H., Sun Q., Zhang X., Jiang P. (2025). Effects of subsurface drip irrigation and nitrogen fertilizer management on N_2_O emissions and forage yield in alfalfa production. Front. Plant Sci..

[37] Zhang J., Li X., Wang J., Yang L., Yang Q., Xiang D., Wan Y., Nevo E., Yan J., Fan Y. (2023). Wild oats offer new possibilities for forage because of the higher nutrition content and feed value. Agronomy.

[38] Mu G. (2025). Resarch on Water–Fertilizer Coupling Effect and Optimization Configuration of Alfalfa Based on CCD–RSM. Master’s Thesis.

[39] Li Y., Ma Z., Liu W., Su M., Wan M., Li Q., Zhang D., Liu J., Wu N. (2025). Effects of vertical deep rotary tillage with organic fertilizer on leaf senescence characteristics and yield of maize in saline soil. Chin. J. Plant Ecol..

[40] Shi D., Jia R., Ye J., Li S., Li C., Li C. (2025). Effects of nitrogen fertilizer postponing and magnesium fertilizer coupling on growth, yield and quality of pineapple. Chin. J. Trop. Crops.

[41] Tian R.R., Wang J.H., Yin M.H., Ma Y.L., Jia Q., Kang Y.X., Qi G.P., Gao Y.L., Jiang Y.B., Li H.Y. (2024). Investigation of the regulatory effects of water and nitrogen supply on nitrogen transport and distribution in wolfberry fields. Front. Plant Sci..

